# Discharged Soldiers as Male Attendants

**Published:** 1917-12-08

**Authors:** 


### Discharged Soldiers as Male Attendants.

The Birmingham Board of Guardians reports that difficulty is experienced from time to time in obtaining suitable male attendants for the different classes of inmates of their institutions. Recently it has been found possible to appoint two discharged soldiers to this position. Generally speaking, however, discharged soldiers are unsuitable for such appointments, owing to their lack of institutional experience and training. The House Committee of the Board feels that considerable advantage would accrue if some arrangement could be set up for the training of these men to fill these positions. It is suggested that if the proposal be approved, the Ministry of Pensions should be asked to regard any discharged soldiers undertaking such preparatory work as soldiers undergoing special training and continue their pensions while they are being trained. The matter has been referred to a special sub-committee to investigate and report.

